# Report of Cases Treated in the Philadelphia Dispensary, for June, 1839

**Published:** 1839-07-27

**Authors:** 


*Report of Cases treated in the Philadelphia Dispensary, for June*, 1839.

Dr. Knight reports, of abortion one case, chronic bronchitis one, contusion one, dysentery four, erysipelas one, intermittent fever one, remittent fever four, gastritis one, chronic hydrocephalus one, ophthalmia two, wound of scalp one, worms one—nineteen cures, one death—nine remain under treatment.

The case of death reported, occurred in a child who was represented as having had hydrocephalus from birth. He had been unwell for several days, and had had convulsions the evening previous to call. Strabismus first supervened about the time of call. Nothing had passed from the bowels for two weeks, notwithstanding the use of various purgatives. Croton oil, however, succeeded in procuring stools. Leeches and cold applications to the head were used, but without any apparent benefit, and the patient died on the sixth day.

Owing to the impatience of friends, only an imperfect autopsy was made sixteen hours after death.

Head large; bones of cranium not separated, but so thin as to be diaphanous. The meninges were neither thickened nor injected; the convolutions were completely unfolded; the ventricles contained ℥iij. of a clear, transparent fluid. The brain presented the appearance of a membrane, which, in many places, was not thicker than the rind of an orange, and was completely softened. The tenia semilunaris, thalami and corpora striata, were in nothing remarkable. The septum lucidum was almost destroyed. Notwithstanding the enormous effusion, and the almost membranous expansion of the brain, the patient's intelligence was as good as is usual with children of his age. It would hence appear that mechanical causes, giving an unnatural form to the brain, do not necessarily interfere with the operations of the mind, provided the deformity be gradually induced.

Dr. Evans reports one case of bronchitis, one of cholera morbus, one of chorea, five of diarrhœa, two of dysentery, one of dyspepsia, one of intermittent fever, one of nebula, two of phthisis, one of pneumonia, one of prolapsus uteri, two of scarlatina, one of worms—20 cases—fifteen cured, three died, (two of phthisis, one of diarrhœa,) three sent to hospital.

Dr. Patterson reports one mammary abscess, one asthma relieved, one apthæ, one cynanche tonsilaris, two cholera infantum, (one died,) two dysentery, five diarrhœa, (one died,) one dyspepsia, one dentition, one delirium tremens, one bilious fever, two remittent fever, two gastritis, (one died, one sent to hospital,) one hepatitis, one hydrocephalus, (died,) one laryngitis, one marasmus, (died,) one metritis, one neuralgia, six pertussis, two phthisis, (one died, one relieved,) one procidentia uteri, (relieved,) two rheumatism, one sprain, one scarlatina, one enlarged tonsils, one ulcer—forty-two cases—thirty-two cured, three relieved, one sent to hospital, and six died.

Dr. Boyer reports one bronchitis, one cachexia, one catarrh, two cephalalgia, one cholera infantum, one concussion of brain, one colic, one diarrhœa, one dysentery, five remittent fever, one hydrarthrosis, one ophthalmia, one pleurisy, one prolapsus ani, three rheumatism—twenty-two cases—twenty cured, one relieved, and one removed. Besides these, there were sixty-three cases prescribed for during the month at the consultation office of the dispensary—twenty-five males, and thirty-eight females—the results of which are unknown. The diagnosis was as follows: chronic bronchitis two, catarrh two, cephalalgia three, cholera morbus two, cholera infantum one, constipation three, contusion three, dentition one, diarrhœa two, dyspepsia four, dysentery one, dysury one, enuresis one, ganglion one, gastralgia one, hæmorrhoids one, hydropericarditis one, leucorrhœa one, derangements of menstruation three, neuralgia one, ophthalmia three, otitis one, pertussis three, pleurisy one, pregnancy one, psoriasis one, rheumatism, enlargement of the spleen one, scrofula two, tinia capitis three, tonsilitis one, ulcer one, vomiting one, worms one.

The previous history of one of the cases of cephalalgia may be worth detailing. The patient is forty years of age, has been a widow nine years, and is the mother of three children, the youngest about seventeen years of age. She was very well after her last accouchement till the third day, when, in consequence of a slight cold, the lochia, previously abundant, ceased suddenly. She felt only slight inconvenience from this, and was up in three weeks. The lochia never returned, nor has she menstruated since. She has no leucorrhœa, either constant or periodical, no symptoms of prolapsus, no vicarious discharge, nor has her health been otherwise disturbed than by a headach recurring without regularity, and accompanied with nausea, and vomiting of a glairy mucous. It is readily amenable to treatment, yielding generally within twelve hours.

The point of interest here is the cessation of the menses at the early age of twenty-three, one year sooner than in any of the cases recorded by Velpeau.

